# Understanding Acid Lability of Cysteine Protecting Groups

**DOI:** 10.3390/molecules18055155

**Published:** 2013-05-06

**Authors:** Iván Ramos-Tomillero, Lorena Mendive-Tapia, Miriam Góngora-Benítez, Ernesto Nicolás, Judit Tulla-Puche, Fernando Albericio

**Affiliations:** 1 Institute for Research in Biomedicine (IRB Barcelona), Baldiri Reixac 10, 08028-Barcelona, Spain; E-Mails: ivan.ramos@irbbarcelona.org (I.R.-T.); lorena.mendive@irbbarcelona.org (L.M-T.); miriam.gongora@irbbarcelona.org (M.G.-B.); judit.tulla@irbbarcelona.org (J.T.-P.); 2 CIBER-BBN, Networking Centre on Bioengineering, Biomaterials and Nanomedicine, Baldiri Reixac 10, Barcelona 08028, Spain; 3 Department of Organic Chemistry, University of Barcelona, Martí i Franqués 1-11, Barcelona 08028, Spain; E-Mail: enicolas@ub.edu; 4 School of Chemistry and Physics, University of KwaZulu-Natal, Durban 4001, South Africa

**Keywords:** acid lability, benzyl, carbocation stability, Cys protecting groups, diphenylmethyl (Dpm), peptide synthesis

## Abstract

Cys-disulfide bonds contribute to the stabilization of peptide and protein structures. The synthesis of these molecules requires a proper protection of Cys residues, which is crucial to prevent side-reactions and also to achieve the correct Cys connectivity. Here we undertook a mechanistic study of a set of well-known acid-labile Cys protecting groups, as well other new promising groups, in order to better understand the nature of their acid-lability. The stability of the carbocation generated during the acid treatment was found to have a direct impact on the removal of the protective groups from the corresponding protected Cys-containing peptides. Hence a combination of steric and conjugative effects determines the stability of the carbocations generated. Here we propose diphenylmethyl (Dpm) as a promising protecting group on the basis of its intermediate relative carbocation stability. All the optimized geometries and energies presented in this study were determined using a B3LYP/6-31G(d,p) calculation. The results discussed herein may be of broader applicability for the development of new protecting groups.

## 1. Introduction

Cysteine (Cys) is one of the key amino acids used by Nature to construct the most important biomolecules. From a chemical point of view, Cys, through its amine and carboxylic functions, forms part of the peptide backbone and, thanks to the thiol side chain, forms intra- and inter-molecular disulphide bridges, which contribute to the stabilization of peptide and protein structures. It is thus not surprising that others and we have devoted much effort to the development of a large number of Cys protecting groups [1].

In addition to developing Cys protecting groups that are removable by enzymes and thiols [2,3], our group seeks to achieve an acid-labile group that is stable in the presence of a low concentration of TFA (<25%) and labile in 60–90% TFA, conditions that can be considered mild. At present, the *p*-methoxybenzyl (Mob) group is the one that most closely resembles these features [4]. However, even though Harris and coworkers showed that the Mob group could be removed under rather mild conditions [5], the removal of this group generally requires the maximum amount of TFA, several hours, and some heating, all of which can jeopardize the syntheses of complex multi Cys-containing peptides.

We recently described a new set of Cys protecting groups with the above-mentioned characteristics, namely diphenylmethyl (Dpm) [6], 2,6-dimethoxybenzyl, and 4-methoxy-2-methylbenzyl [7]. Given the easier accessibility of Dpm, this group is recommended for filling the gap in the arsenal of acid-labile Cys protecting groups. Considering Dpm, Cys has three distinct compatible acid-labile protecting groups: Mmt or Trt, removable with a low concentration of TFA; Dpm, stable to low concentrations of TFA, but removable at high concentrations; and *p*-methylbenzyl (Meb), stable to TFA and removable by HF. The two first options are suitable for Fmoc chemistry while the third is appropriate for Boc chemistry. With Dpm, Cys matches the same map of protecting groups available for the other amino acids [Trt- (low TFA), *t*-Bu- (high TFA), Bzl/cHex- (HF) based protecting groups for Lys, Tyr, Ser/Thr, or Glu/Asp]. Given the intriguing results obtained in our previous study, here we undertook a mechanistic study in an attempt to further clarify them.

## 2. Results and Discussion

Most protecting groups and linkers used in solid-phase peptide synthesis (SPPS) [8,9] are based on benzyl (Bzl), diphenylmethyl (Dpm), and triphenylmethyl (Trityl, Trt) structures. Given that these classes of protecting groups are removed through a carbocation intermediate, the stability of the latter has a direct effect on the lability of the corresponding protecting group. Several factors are proposed to contribute to the stability of the carbocation generated, including steric and field-inductive effects, in addition to the direct electronic effects caused by any substituents attached to the carbocation center. The introduction of extra phenyl groups stabilizes the carbocation through π-type delocalization by resonance, resulting in an increase in carbocation stability as follows ArCH_2_^+^ < Ar_2_CH^+^ < Ar_3_C^+^ [10,11].

Furthermore, the stability of the cationic species is determined not only by the electronic character of any substituents present, but also by their relative position in the aromatic ring. Thus, the electron-donating substituents in *ortho-* and *para-*positions should further stabilize the cationic system through a resonance effect and have a much smaller influence in the *meta-*position [12].

### 2.1. Experimental Data

Using the tripeptide Fmoc-Ala-Cys(PG)-Leu-NH_2_ built on a Sieber amide resin as a model, we recently evaluated the TFA lability of two scaffolds—Bzl and Dpm—in the presence of 2.5% of H_2_O and 2.5% of triisopropilsilane (TIS) as scavengers [7]. Table 1 shows the results for Dpm and Bzl derivatives, respectively.

**Table 1:** TFA-lability study of the tripeptides with Dpm and Bzl scaffolds [7].

	Abbr.	Protecting group	TFA (%)	[Peptide] (mM)	T (°C)	Reaction time	Deprotected Cys (%)
**1**	4,4′-diMeODpm	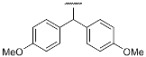	10	1	25	5 min	100
				10			92
**2**	4,4′-diMeDpm	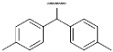	20	1	25	5 min	92
				10			
							70
				1		30 min	100
				10			100
**3**	Dpm	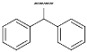	60	1	25	1 h	100
				10			92
**4**	9-F	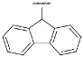	95	1	25	1 h	0
				10			0
**5**	2,6-diMeO-4-MeBn	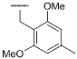	20	1	25	30 min	100
				10			100
**6**	2,4-diMeOBn	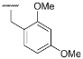	20	1	25	5 min	70
				10		5 min	10
				1		30 min	100
				10		30 min	44
**7**	2,6-diMe-4-MeOBn	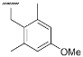	20	1	25	30 min	100
				10			86
**8**	2,6-diMeOBn	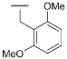	50	1	25	1 h	100
				10			96
**9**	4-MeO-2-MeBn	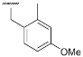	50	1	25	1 h	100
				10			95
**10**	Mob	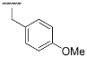	95	1	25	2 h	35
				10			26
				1	40		100
				10			94
**11**	TMeb	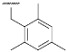	95	1	25	1 h	21
				10			14
**12**	biPh	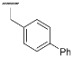	95	1	25	1 h	0
				10			0
**13**	2-MeOBn	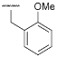	95	1	25	1 h	0
				10			0

The following conclusions can be drawn from these results: (i) concentration, reaction time, and temperature affect the lability of the protecting group; (ii) optimum results are achieved only when using scavengers to trap the carbocation and to shift the equilibrium towards the unprotected species [13]; and (iii) the introduction of an electron-donating group (e.g., -OMe or -Me) in an aryl moiety implies a subsequent increase in its acid lability, which in turn depends on the strength of the electron-donating character. For instance, the series of derivatives **1**, **2**, **3**, and **5**, **8** (-OMe, -Me, -H) are illustrative examples of this behaviour.

In contrast, the position of the substituents has a more dramatic effect on the lability of the protecting group. Thus, based on the comparison of either pairs **6 ***vs.*** 8** or **10*** vs. ***13**, it can be concluded that *para*-substituents have a greater effect on acid lability than *ortho-* ones. We consider this is clearly remarkable in the second pair, where **10** is partially removable at 25 °C and the *ortho-*regioisomer is totally stable.

### 2.2. Computational Study

In order to explain the variations in lability of these protecting groups as well as that of others described in the literature [methoxytrityl (Mmt), methyltrityl (Mtt), trityl (Trt), xanthenyl (Xan)] [4,14,15,16,17,18], here we performed a computational study to analyze the stability of the corresponding carbocation species. This measure of stability was expressed by the total electronic energy difference (Δ*E*) between the carbocation (R^+^) and its neutral precursor (R-H) in the following reaction [19,20]:

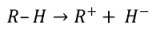



Thus, Δ*E* was determined for each protecting group using the following expression:

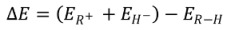

where the energetic terms 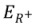 ,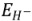, and *E_R_*_-*H*_ were obtained from the calculation of the minimum of energy of the corresponding drawn structures. No imaginary frequencies for minima provide a control that the stationary point localized is correct. Moreover, apart from the Δ*E* calculation, Δ*E* + *ZPE*, ∆*H* and ∆*G* were determined. The ∆*E* + *ZPE* term is the electronic energy for each process, taking into account the *ZPE* (Zero Point Energy) correction, and ∆*H* and ∆*G* are the corresponding enthalpy and Gibbs energy, respectively. The computational results for Trt, Dpm and Bzl protecting groups are reported in Table 2, Table 3, Table 4, respectively. No solvation effects were considered, and the values are given in kcal·mol^–^^1^.

In general, the computational results of the carbocation stabilities –and consequently the lability of the protecting groups—are consistent with the previously reported experimental results and also with the trends discussed above. Thus, an increase in stability was found to be associated with both the number and type of electron-donating substituents.

**Figure 1:**
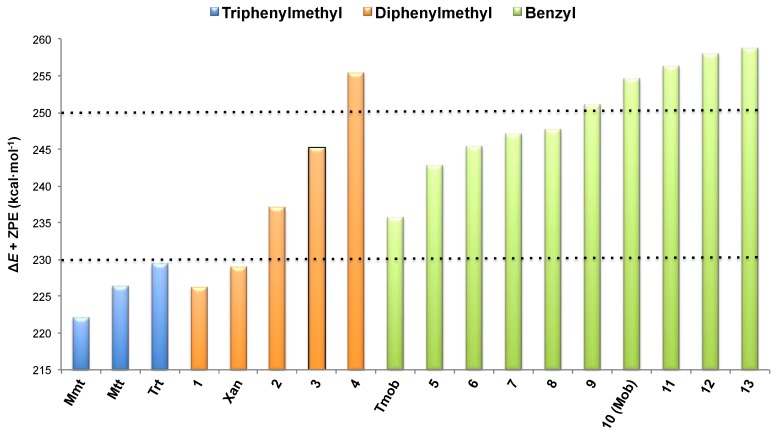
Δ*E* + ZPE values for each scaffold group.

Depending on the Δ*E* calculated, three regions limited by dotted lines were distinguished (Figure 1). In general terms, we defined highly- (up to 230 kcal·mol^−^^1^), intermediate- (from 230 to 251 kcal·mol^−^^1^) and low-acid labile (upper to 251 kcal·mol^−^^1^) intervals.

**Table 2:** Trt derivatives.

	Δ*E*	Δ*E *+ ZPE	Δ*H*	Δ*G*
**Mmt**	228.3	222.0	223.2	217.4
**Mtt**	232.9	226.4	227.6	221.9
**Trt**	235.8	229.4	230.6	224.7

**Table 3:** Dpm derivatives.

	Δ*E*	Δ*E* + ZPE	Δ*H*	Δ*G*
**1**	232.2	226.2	227.4	221.5
**Xan**	235.2	228.9	230.1	223.6
**2**	243.6	237.1	238.3	232.4
**3**	251.7	245.2	246.5	240.6
**4**	262.5	255.5	256.9	249.2

**Table 4:** Bzl derivatives.

	Δ*E*	Δ*E *+ ZPE	Δ*H*	Δ*G*
**Tmob**	241.9	235.8	236.9	230.5
**5**	249.1	242.8	243.9	237.6
**6**	251.8	245.4	246.6	239.5
**7**	253.4	247.1	248.2	241.9
**8**	254.0	247.7	248.8	242.5
**9**	257.7	251.1	252.4	245.0
**10 (Mob)**	261.0	254.6	255.7	249.5
**11**	263.2	256.3	257.5	250.5
**12**	264.5	258.0	259.1	253.2
**13**	265.4	258.8	260.1	252.9

A close examination of Dpm structures indicates that a delicate balance of steric and conjugative effects determines their corresponding stability. Regarding the stability of the carbocation, the robustness of **4** can be explained by the inherent anti-aromaticity caused by its 4n cyclic π-framework; in contrast, the high lability of **Xan** is attributed to the π-delocalization around the tricyclic system, which confers aromaticity to the carbocation.

**Figure 2:**
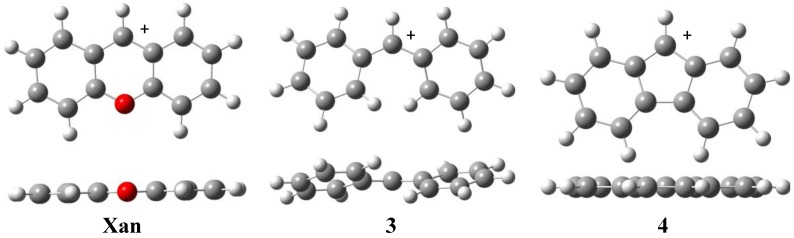
Optimized geometries of the carbocations derived from Xan, 3 and 4, respectively.

The optimized structures for Dpm scaffolds (**1**, **2** and **3**) show a lack of planarity; the phenyl rings are twisted out of plane in order to minimize interactions between *ortho*-substituted hydrogen atoms. Nonetheless, the calculated structures for **Xan** and **4** retain planarity because of their restricted Ph-Ph bond (Figure 2). This observation was previously reported by Hoffman [10] and Lee-Ruff [21].

On the basis of these results, we confirm that Dpm shows intermediate relative carbocation stability (Figure 1) and propose it as a promising protecting group to fill the current gap. This finding is in accordance with our previous study [7], which demonstrated experimentally the compatibility of Dpm with the commonly applied acid-labile groups Trt and Mmt. Thus, in a three Cys-containing hexapeptide protected with Dpm, Trt and Mmt groups, respectively, Trt and Mmt were fully removed on solid-phase by applying a mixture of 10% TFA and 2.5% TIS in DCM, while Dpm remained unaltered. In contrast, the selective removal of Mmt *vs.* Trt was attempted using a range of cleavage conditions. However, as a result of the similar acid lability of these two protecting groups, as determined in the present computational study, the safety window is so narrow that exclusive removal of Mmt in the presence of Trt was unsuccessful, and therefore not of general applicability.

**Figure 3:**
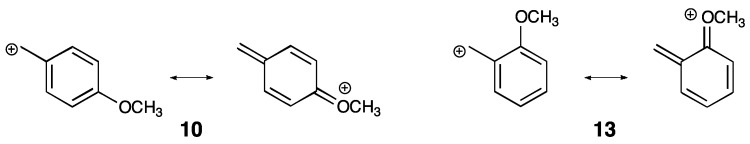
Comparison of *para*- (10) and *ortho-* (13) Bzl-like resonance structures.

The computational energy values calculated for **6*** vs. ***8** or **10*** vs. ***13** corroborate the previous experimental results regarding the lower energy of *para*-methoxy-containing compounds compared to the *ortho*-methoxy-containing analogs (Figure 3). We ascribe this observation to the fact that the resonance structure of *para*-methoxy derivatives with the positive charge on the oxygen atom is symmetrically more stabilized than the corresponding *ortho*-methoxy structures.

## 3. Experimental

All geometries and energies reported in this study were calculated using the B3LYP density functional theory, as implemented in the Gaussian 03 program package [22]. Geometry optimizations were performed using the 6-31G(d,p) basis set. Vibrational frequencies were calculated in order to confirm that a minimum energy had been obtained.

## 4. Conclusions

In conclusion, here we confirm that proper combination of computational studies and experimental work can be extremely useful for the design of new protecting groups. The results reported here may also be of a broader applicability for the development of protecting groups for other chemical functions and linkers for SPPS.
